# Barriers to the donation of living kidneys for kidney transplantation

**DOI:** 10.1038/s41598-022-06452-9

**Published:** 2022-02-14

**Authors:** Kyungok Min, Tai Yeon Koo, Young Hui Hwang, Jaeseok Yang

**Affiliations:** 1 Transplantation Center, Seoul National University Hospital, Seoul, Republic of Korea; 2 Department of Nephrology, Seongnam Citizens Medical Center, Seongnam, Gyenggi-do Republic of Korea; 3 Department of Nursing, University of Ulsan, Ulsan, Republic of Korea; 4 Division of Nephrology, Department of Internal Medicine, Yonsei University College of Medicine, Severance Hospital, 50 Yonsei-ro, Seodaemun-gu, Seoul, 03722 Republic of Korea

**Keywords:** Health care, Nephrology

## Abstract

Since the waiting time for deceased donor kidney transplantation continues to increase, living donor kidney transplantation is an important treatment for end stage kidney disease patients. Barriers to living kidney donation have been rarely investigated despite a growing interest in the utilization of living donor transplantation and the satisfaction of donor safety. Here, we retrospectively analyzed 1658 potential donors and 1273 potential recipients who visited the Seoul National University Hospital for living kidney transplantation between 2010 and 2017 to study the causes of donation discontinuation. Among 1658 potential donors, 902 (54.4%) failed to donate kidneys. The average number of potential donors that received work-up was 1.30 ± 0.66 per recipient. Among living donor kidney transplant patients, 75.1% received kidneys after work-up of the first donor and 24.9% needed work-up of two or more donors. Donor-related factors (49.2%) were the most common causes of donation discontinuation, followed by immunologic or size mismatches between donors and recipients (25.4%) and recipient-related factors (16.2%). Interestingly, withdrawal of donation consent along with refusal by recipients or family were the commonest causes, suggesting the importance of non-biomedical aspects. The elucidation of the barriers to living kidney donation could ensure more efficient and safer living kidney donation.

## Introduction

Kidney transplantation is the best renal replacement therapy that can improve the survival and quality of life of patients with end-stage kidney disease (ESKD). However, the number of deceased kidney donation is limited as compared to the number of ESKD patients; therefore, the waiting time for deceased donor kidney transplantation (DDKT) is prolonged, especially in Asian countries where the donation rate of deceased donor kidneys is much lower than that in Western countries. Living donor kidney transplantation (LDKT) is an important alternative to ESKD patients due to severe organ shortage.

The number of living kidney transplants in Korea has been continuously increasing from 796 cases in 2010 to 1432 cases in 2020^1^. In Korea, the National Health Insurance Service (NHIS) serves as the insurer, and it is mandatory for an individual to obtain the national medical insurance. The major sources of NHIS funding includes contribution from those who are insured. The Organ Transplantation Law of Korea requires a recipient to pay for the cost of donor work-up before transplantation and donor nephrectomy. Since 2010, ESKD patients pays 10% of medical costs, and the 90% remainder is paid by the NHIS. Therefore 90% of the cost of recipient work-up before transplantation are paid by the NHIS and 10% by recipient. Although 100% of the cost of donor work-up before transplantation are paid by recipient, 80% of this cost is returned after kidney donation by NHIS. Some low-income patients are exempt from the 10% of medical cost. If donors are discriminated at workplace after donation, government will charge a fine to the company. The Organ Transplantation Law of Korea applies equally to both Korean and foreigners at the living donor transplantation. According to the World Health Organization Global Database On Donation And Transplantation statistics in 2019, Korea ranked 3rd in the world for the highest number of living donor kidney transplantations^2^, indicating that living donor evaluation is essential in Korea.

Kidney donation does not significantly affect quality of life, kidney function, or survival of living donors^3^. Although kidney donation is based on spontaneity, the physical risks from procurement of healthy kidneys and the uncertainty of future health conditions must be endured^4^. Since living donation is dependent on voluntary, free will of healthy people, safety of living donors is the most important concern. Therefore, potential donors should receive objective information with respect to the biomedical as well as the non-biomedical aspect.

However, the criteria for living kidney donors are variable among different transplantation centers and periodic follow-up of kidney donors after donation is poor in Korea^5^. Furthermore, an aggravating discrepancy between the need and supply has made the criteria for living kidney donors more flexible over time^6,7^.

In this situation, it is vital to understand the current barriers to successful kidney donation and make an effort to overcome them for safe and efficient kidney donation. The Live Donor Community of Practice (LDCOP) within the American Society of Transplantation hosted a Consensus Conference on Best Practices in Live Kidney Donation to identify knowledge gaps that could affect LDKT with focus on the systematic barriers to LDKT^8^. However, there are limited studies exploring the causes of kidney donation discontinuation from living kidney donors despite a growing interest in this topic^9,10^. There have been few studies on living kidney donors in Asian countries where LDKT is dominant over DDKT^5^. Therefore we analyzed the factors that affect donor access to verify appropriateness of donor selection, and identified the causes of donation discontinuation from living kidney donors in Korea.

## Results

### Clinical characteristics of potential living donors and recipients

**Figure 1:**
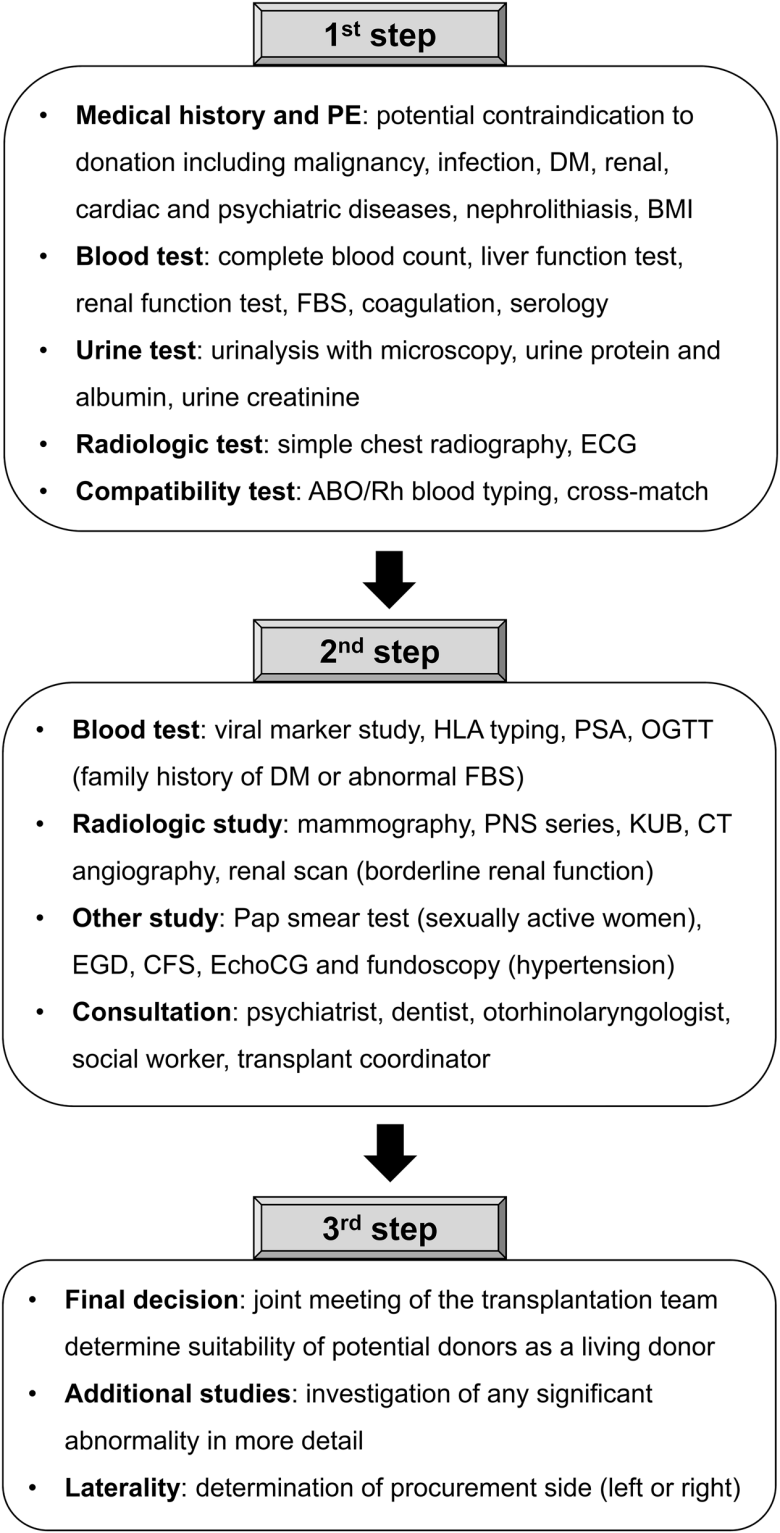
The stepwise screening processes for potential living kidney donors. The evaluation for potential living kidney donors consists of three steps. BMI, body mass index; CFS, colonofiberscopy; DM, diabetes mellitus; ECG, electrocardiogram; EchoCG, echocardiography; EGD, esophagogastroduodenoscopy; FBS, fasting blood sugar; KUB, kidney, ureter, and bladder; OGTT, oral glucose tolerance test; PE, physical examination; PNS, paranasal sinus.

A total of 1658 potential living kidney donors received cross-match tests with corresponding 1273 potential kidney transplant recipients and received a stepwise donor evaluation (Fig. 1). The mean age of the potential donors was 44.9 ± 11.4 years and 50.8% were females (n = 838). Related donors and unrelated donors were 1,076 (64.9%) and 578 (34.9%), respectively. Spouse donors were most common (n = 467, 28.2%). A donor exchange program provided 4 donors (0.2%). The time from the initial work-up to living donor kidney transplantation operation was 194.0 ± 173.0 days. Other clinical characteristics of potential donors are summarized in Table 1. After transplantation, all donors are alive until 31, July 2021.

**Table 1:** Clinical characteristics of potential donors.

	N = 1658	Mean ± SD or number (%)
Age (years)		44.9 ± 11.4
Sex (female)		838 (50.8)
BMI (kg/m^2^)		24.0 ± 3.3
BSA (m^2^)		1.7 ± 0.2
Relation to a potential recipient	Parent	344 (20.7)
	Offspring	273 (16.5)
	Sibling	372 (22.5)
	Other related donors	87 (5.2)
	Spouse	467 (28.2)
	Other unrelated donors	111 (6.7)
	Donor exchange	4 (0.2)

BMI, body mass index; BSA, body surface area; SD, standard deviation.

The mean age of the potential recipients was 43.8 ± 16.2 years and 42.9% were females (n = 711). Glomerulonephritis (35.0%) was the most common cause of ESKD, followed by diabetes mellitus (DM) (21.5%). Two hundred fifty-one patients (19.7%) planned to proceed to pre-emptive transplantation and 788 (61.9%) patients were under hemodialysis. Other clinical characteristics of potential recipients are summarized in Table 2.

**Table 2:** Characteristics of potential recipients.

	N = 1273	Mean ± SD or number (%)
Age (years)		43.8 ± 16.2
Sex (female)		711 (42.9)
BMI (kg/m^2^)		22.6 ± 4.1
BSA (m^2^)		1.7 ± 0.6
Cause of ESKD	Glomerulonephritis	446 (35.0)
	Diabetes mellitus	274 (21.5)
	ADPKD	107 (8.4)
	Hypertension	61 (4.8)
	Reflux nephropathy	26 (2.0)
	Miscellaneous	154 (12.1)
	Unknown	205 (16.1)
RRT before transplantation	Pre-emptive	251 (19.7)
	Hemodialysis	788 (61.9)
	Peritoneal dialysis	210 (16.5)
	Unknown	24 (1.9)

ADPKD, autosomal dominant polycystic kidney disease; BMI, body mass index; BSA, body surface area; ESKD, end stage kidney disease; RRT, renal replacement therapy; SD, standard deviation.

### Proceed to kidney transplantation after work-up of potential living donors

The average number of potential donors that received donor work-up was 1.30 ± 0.66 per recipient. The number of potential donors that received donor work-up in recipients with positive cross-match results or donor specific antigen (DSA) (2.10 ± 1.50) was higher than that in recipients without positive cross-match results and DSA (1.56 ± 0.86, *P* < 0.001), and that in recipients with ABO-incompatible donors (1.65 ± 0.89, *P* < 0.001).

**Figure 2:**
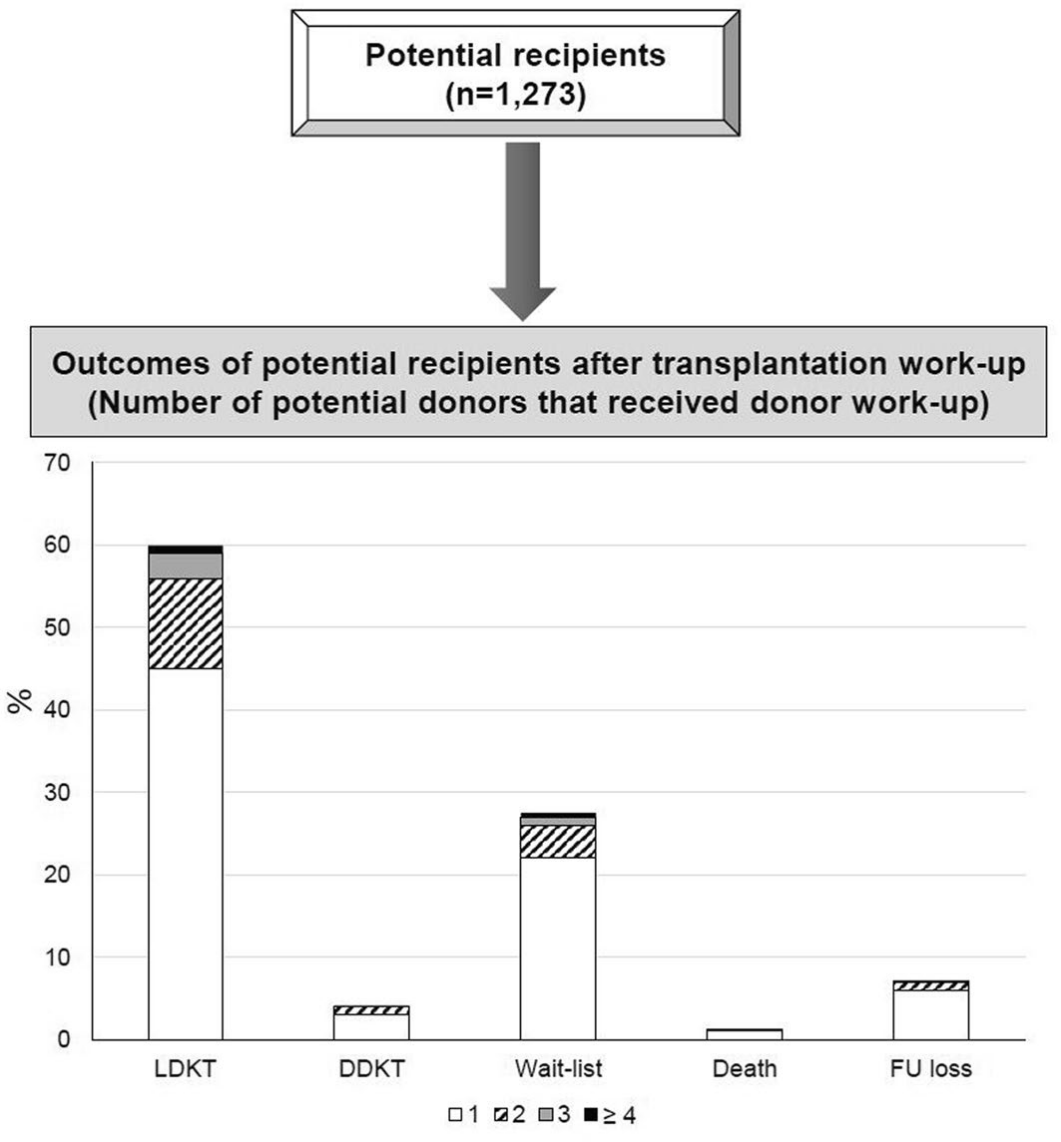
Number of potential donors per recipient that received donor work-up and outcomes of potential recipients after transplantation work-up. In total, 1273 potential recipients were cross-matched with 1658 potential donors. After transplantation work-up, potential recipients received LDKT (n = 756) or DDKT (n = 59) were still on the waitlist for DDKT (n = 347), died (n = 14), or were lost to follow-up (n = 97). DDKT, deceased donor kidney transplantation; FU, follow-up; LDKT, living donor kidney transplantation.

After work-up, 756 donors among the 1658 potential living donors succeeded in donating their kidneys (45.6%) and living donor kidney transplantation was successfully performed for 756 among 1273 recipients (59.4%). Of the patients who received living donor kidney transplantation, 75.1% received kidneys after work-up of the first potential donor and 24.9% needed work-up of two or more potential donors (Fig. 2). ABO blood group-incompatible living donor transplantation was performed in 103 cases (13.6%). After donation discontinuation to receive living donor kidneys, 59 patients (4.6%) received deceased donor kidney transplantation and 347 patients were still on the waitlist for deceased donor kidney transplantation (27.3%). Two or more potential donors were assessed but failed to donate kidneys in 82 patients (20.2%) among a total of 406 DDKT and wait-list patients. Fourteen patients died (1.1%) and 97 patients (7.6%) were lost to follow-up.

### Causes of donation discontinuation of potential living donors

Among 1,658 potential living kidney donors, 902 (54.4%) failed to donate kidneys. Causes of kidney donation discontinuation are summarized in Table 3. Donor-related factors (n = 444, 49.2%) were the most common causes. Recipient-related factors and mismatches between donors and recipients led to kidney donation discontinuation in 146 (16.2%) and 229 (25.4%) cases, respectively. According to the stepwise donor evaluation process, 687 (76.2%) potential donors did not fulfil the criteria of living kidney donors at the first step and donation discontinuation occurred in 98 (10.9%) and 117 (13.0%) patients in the second and third step, respectively.

**Table 3:** Causes of discontinuation to donate living kidneys.

Causes of donation discontinuation	N = 902	Number (%)
Donor-related factors		444 (49.2%)
	Low eGFR	17 (1.9%)
	Diabetes mellitus	37 (4.1%)
	Hypertension	41 (4.4%)
	Glomerulonephritis	25 (2.8%)
	Obesity	20 (2.2%)
	Malignancy	16 (1.8%)
	Stone	14 (1.6%)
	Old age (≥ 60 years)	8 (0.9%)
	Genetic disease	7 (0.8%)
	Cardiovascular disease	6 (0.7%)
	Anatomical problems	5 (0.6%)
	Viral hepatitis	5 (0.6%)
	Major psychiatric disorder	3 (0.3%)
	Unmarried status or pregnancy	9 (1.0%)
	Presence of better donor	61 (6.7%)
	Failure of KONOS approval	48 (5.3%)
	Withdrawal of donation consent	113 (12.5%)
	Miscellaneous	9 (1.0%)
Recipient-related factors		146 (16.2%)
	Cardiovascular disease	22 (2.4%)
	Malignancy	28 (3.1%)
	Infection	8 (0.9%)
	Neurogenic bladder	2 (0.2%)
	High risk of recurrent renal disease	4 (0.4%)
	Registration for multiorgan deceased donor transplantation	5 (0.6%)
	Deceased donor transplantation during work-up	6 (0.7%)
	Death during work-up	8 (0.9%)
	Improved renal function	2 (0.2%)
	Refusal by recipients	53 (5.9%)
	Miscellaneous	8 (0.9%)
Mismatch between donors and recipients		229 (25.4%)
	Positive cross-match test results	192 (21.2%)
	Positive DSA with negative cross-match test results	6 (0.7%)
	HLA mismatching	10 (1.1%)
	ABO-incompatibility	14 (1.6%)
	Recipient BSA/donor BSA > 1.3	7 (0.8%)
Other causes		83 (9.2%)
	Decline by family	39 (4.3%)
	Delay	6 (0.7%)
	Transfer or follow-up loss	38 (4.2%)

BSA, body surface area; DSA, donor specific antigen; eGFR, estimated glomerular filtration rate by MDRD equation; HLA, human leukocyte antigen; KONOS, Korean Network for Organ Sharing.

Among donor-related factors, various medical disorders such as DM, hypertension with target organ damage, cardiovascular disease, viral hepatitis and malignancy (n = 105) were common barriers to donation as predicted. However, withdrawal of donation consent was the most common cause among donor-related causes (n = 113). The third most common cause among donor-related causes was the presence of better donors among multiple potential donors (n = 61). The Korean Network for Organ Sharing (KONOS) did not approve living donation in 48 unrelated donors since their documents could not sufficiently prove their altruistic and voluntary donation without illegal incentives including financial incentives. Glomerulonephritis and genetic diseases that potential recipients shared, were barriers to donation in 25 and 7 donors, respectively. The number of donation discontinuation due to a low estimated glomerular filtration rate (eGFR) (eGFR < 60 ml/min) (n = 17) and other anatomical causes (n = 5) as a cause of donation discontinuation was relatively small. As similar to other transplant centers^11,12^, arterial or venous multiplicity of potential donors was relative contraindication in our center, and transplant surgeons decided on operable cases. Five donors could not donate their kidneys due to the followings reasons; both double renal veins with left sided inferior vena cava, left retroaortic renal vein and right multiple renal veins, right renal artery aneurysm with calcification and left multiple renal arteries with diffuse calcification and atherosclerosis, right renal atrophy, and medullary sponge kidney. In our center, obesity (BMI > 30 kg/m^2^) is a contraindication to kidney donation. Twenty potential donors failed to donate their kidneys due to obesity. Old age were barrier to donation in 8 donors. Two donors, which were in their sixties (60 years, 62 years), could not donate their kidneys because there were alternative young donors among multiple potential donors. The remaining 6 donors were seventies, and advanced age (older than 70 years) were contraindication to kidney transplantation in our center until 2015.

Among recipient-related factors, refusal by recipients was the most common cause (n = 53). Among medical complications of recipients, presence of cardiovascular diseases (n = 22) and malignancy (n = 28) were common causes of donation discontinuation. When the recipients developed other organ failure, we recommended registration for multiorgan deceased donor transplantation (n = 5). Six recipients performed DDKT during preparation of LDKT. Neurogenic bladder is a condition associated with high incidence of urinary tract infection and graft function after transplantation, and was previously considered to be a relative contraindications. In our center, neurogenic bladder caused donation discontinuation in 2 cases.

There were immunologic mismatches (n = 222) and size mismatches (n = 7) in mismatch-related causes. Positive cross-match test results (n = 192) were the most common causes of donation discontinuation among mismatch-related causes. ABO-incompatibility itself or very high titer of anti-ABO antibodies (> 1:1024) were causes of donation failure in 14 cases. When HLA mismatch number were different among multiple donors, we selected the donor with the smallest number of HLA mismatch (n = 10). Donor /recipient BSA ratio is a reliable indicator of nephron dosing^13^. Donor/recipient BSA ratio > 1.3 was a contraindication to kidney transplantation in our center. Seven potential donor failed to donate their kidneys due to size mismatches.

There were also other factors (n = 83, 9.2%) that caused donation discontinuation. The donor's primary guardian, for example, parents or spouse of potential donors, did not consent in 39 cases. Transfer to other hospitals or follow-up loss in the middle of work-up occurred in 38 cases.

### Frequency and causes of donation discontinuation according to relationship to potential recipients

Donation discontinuation rates were different according to relationship to potential recipients (*P* < 0.001, Table 4). Donation discontinuation rate was lowest in potential parent donors (136 among 344, 39.5%) and was highest in other unrelated donors (92 among 111, 83.9%). When causes of donation discontinuation was analyzed according to relationship to potential recipients (Table 4), contribution of mismatch factors to donor discontinuation was relatively low in parent donors (8.1%) and high in spouse donors (36.9%). In unrelated donors except spouse, failure of KONOS approval was the dominant cause of donation discontinuation among donor factors (83.9%). In offspring donors, donor factors (32.2%) contributed to donor discontinuation to less extent than donors with other relationship, while recipient factors (31.0%) did to more extent (Table 4). Interestingly, the major recipient factor for donation discontinuation in offspring donors was parent’s refusal to receive offspring’s kidneys (52.8%). In sibling donors, donor factors markedly contributed to donation discontinuation (62.9%, Table 4). Among donor factors, withdrawal of donation consent (36.9%) was the most common in sibling donors, similarly as in spouse donors (40.6%).

**Table 4:** Frequency and causes of donation discontinuation according to relationship to potential recipients.

Relationship to potential recipients	Frequency of donation discontinuation, Number	Causes of donation discontinuation, Number (%)			
		Donor factors	Recipient factors	Mismatch factors	Other factors
Parent	136	78 (57.4%)	24 (17.6%)	11 (8.1%)	23 (16.9%)
Offspring	171	55 (32.2%)	53 (31.0%)	44 (25.7%)	19 (11.1%)
Sibling	194	122 (62.9%)	18 (9.3%)	41 (21.1%)	13 (6.7%)
Other related donors	49	27 (55.1%)	6 (12.2%)	13 (26.5%)	3 (6.1%)
Spouse	260	106 (40.8%)	40 (15.4%)	96 (36.9%)	18 (6.9%)
Other unrelated donors	92	56 (60.9%)	5 (5.4%)	24 (26.1%)	7 (7.6%)
Total	902	444 (49.2%)	146 (16.2%)	229 (25.4%)	83 (9.2%)

### Clinical outcomes of living donor kidney transplants and donors in our transplant center

Data from Korean Organ Transplantation Registry (KOTRY)^14^ showed that the patient survival rates in LDKT at 1 and 5 years were 98.4% and 96.3%, respectively, and death-censored graft survival rates in LDKT at 1 and 5 years were 98.4% and 94.0%, respectively. In our center, 1-year and 5-year patient survival rates were 97.2% and 95.0%, respectively, and 1-year and 5-year death-censored graft survival rates were 98.2% and 94.1%, respectively.

All living donors are alive and no one had ESKD after kidney donation in our center.

## Discussion

In this study, we followed up with potential living donors after donor evaluation and analyzed the causes of donation discontinuation. A total of 756 donors among 1658 potential living donors succeeded in donating their kidneys (45.6%) and 756 living donor kidney transplantation were performed for 1273 recipients (59.4%). The average number of potential donors that received donor work-up was 1.30 ± 0.66 per a recipient. Among LDKT patients, 75.1% received kidneys after work-up of the first potential donor and 24.9% needed work-up of two or more potential donors. Among 1,658 potential living kidney donors, 902 (54.4%) failed to donate kidneys. Donor-related factors (n = 444, 49.2%) were the most common causes of donation discontinuation. Recipient-related factors and immunologic or size mismatches between donors and recipients caused donation discontinuation in 146 (16.2%) and 229 (25.4%) cases, respectively. Donation discontinuation rates were lowest in parent donors, whereas they were highest in unrelated donors except spouse.

An early study reviewed the consequences of kidney donation on the health of living kidney donors^15^. Living kidney donors did not have a higher risk of death compared to the general healthy population. There was a risk of accelerating eGFR loss, but the ESKD risk was very low^15^. There was no evidence of an increased risk of ESKD for living kidney donors compared to the general population; however, living kidney donors were at an increased risk of ESKD compared to healthy non-donors, although the absolute increase in the ESKD risk attributable to donation was minimal^16–18^. Based on these data, evaluation and minimization of post-donation ESKD risk have been a priority in screening potential living kidney donors^19^. It is essential to balance between the most suitable kidney for the beneficiary and the safety of a single kidney donor for the rest of their life in the donor selection process. From this point of view, it is important to establish the criteria for absolute or relative contraindications about donor selection. The proportions of donors who violated absolute or relative contraindications in the screening process may vary from center to center. A study conducted in Australia and New Zealand found that 767 (26%) of 2932 donors disregarded at least one relative contraindication according to the Caring for Australians with Renal Impairment (CARI) guidelines, 268 patients (9%) proceeded to kidney donation by violation of at least one absolute contraindication, and there was no clear change in donor risk profile over time^20^. Our transplant center have been applying our own absolute or relative contraindications to approve or exclude potential living donors based on AST guideline^21^. However, no living kidney donor who violated the absolute contraindications proceeded to kidney transplantation in this study. Our donor evaluation program consists of three sequential steps to minimize unnecessary testing. The first step is to check the contraindications for living kidney donation and initial education by physician about kidney transplantation and living donation. The second step is to evaluation of the medical condition, psychiatric consultation, social worker interview, education by transplant coordinators and making informed consent. The final, third step is the decision of suitability of potential donors at transplant team meeting. Based on this stepwise approach, 76.2% of all donation discontinuation cases occurred at the first step, suggesting efficiency of our evaluation system. However, most cases of withdrawal of donation consent occurred at the second step, especially psychiatric consultation, and transplant coordinator’s education. Donation discontinuation at the second step could cause economic burden to potential recipient because donor work-up was not covered by public insurance in cases of donation discontinuation in Korea. Therefore, we plan to provide both recipients and their potential donors with psychiatric consultation and transplant coordinator’s education just before the second step, and improve physician education program at first step.

For causes of donation discontinuation, donor-related factors were the most common causes. However, criteria for donor-related factors have been made more flexible, due to severe organ shortage^6,7^. Living donors with the relative contraindications, such as impaired fasting blood glucose, controlled hypertension without evidence of target organ damage, and positive hepatitis B virus surface antigen (HBsAg) without clinical and laboratory evidence of hepatitis, have tried to donate their kidneys. Of course, further long-term studies should confirm safety of these expanded criteria living donors after donation. We excluded five potential donors due to positive HBsAg in this study; however, we have recently changed this policy and have started to accept these donors in recipients with protective anti-HBs antibody. Eight potential donors failed to donate their kidneys due to their advanced age. However, the average age of living kidney donors has steadily increased in Korea with increase in the expected lifespan^1^. Although upper limit of donor age used to be 70, our center has accepted donors by age of 75 years since 2015. Therefore, henceforth, advancing age will not be a cause of donation discontinuation as far as the donors is physically healthy.

Immunologic or size mismatch between donor and recipients were also important causes of donation discontinuation. In parallel, the number of potential donors that received donor work-up in sensitized recipients was higher than that in recipients without DSA or ABO-incompatible donors. Presence of DSA and positive cross-match test results used to contraindications to donation as they can induce hyperacute or severe acute antibody-mediated rejection in sensitized recipients^22^; thus, 22.0% of potential donors failed to donate their kidneys. However, waiting time of sensitized recipients for deceased donor transplantation is also longer than that of wait-listed patients without sensitization^23^. Since living donor kidney transplantation after desensitization led to better patient survival than waiting for deceased donor transplantation in the United States and Korea^24,25^, active application of desensitization to highly sensitized recipients is expected to increase utilization of these mismatched donors. On the other hand, 14 potential donors failed to donate their kidneys due to ABO-incompatibility and high titer of anti-ABO antibodies in this study. However, development of effective desensitization technology has markedly improved outcomes of ABO-incompatible living donor kidney transplantation worldwide^26–29^. The number of ABO-incompatible living donor kidney transplantations in Korea has rapidly increased from 78 cases in 2010 to 709 cases in 2019, which corresponds to 16.4% of all living donor kidney transplantation^9^. In parallel with this trend, our results also include ABO-incompatible living donor transplantation in 103 cases (13.6%) in this study, no longer regarding an ABO-incompatibility as a contraindication to living kidney donation. Ten donors failed to donate their kidneys due to HLA mismatch. The higher degree of HLA mismatch between recipient and donor is associated with worse transplant outcomes^30,31^. We chose the donor with the smallest number of HLA mismatch. Several studies reported that body surface area (BSA) was an important predictor for delayed graft function, acute rejection and graft function after transplantation^32–34^. Based on these data, we applied criteria that BSA of more than 1.3 was relative contraindication in kidney transplantation until 2014. However, we do not consider BSA in the process of transplantation work-up due to severe organ shortage and lesser medical significance.

Recipient-related factors, including malignancy and cardiovascular diseases, also interfered with living donor kidney transplantation. A European guideline recommends malignancy screening in potential living kidney donors and recipients according to the recommendations that apply to the general population and suggests that cases with current or previous cancer be discussed with an oncologist on a case-by-case basis^35^. Although Korea is implementing a nationwide cancer health screening program, living donor kidney transplantation did not proceed due to a recipient’s malignancy (3.1%) in this study. Therefore, we need periodic and more detailed screening programs for malignancy in ESKD or advanced chronic kidney disease patients in addition to a national screening program.

Interestingly, withdrawal of donation consent (12.5%) was the most common cause of donation discontinuation as a single donor-related cause, especially in sibling and spouse donors. Our interview with donation withdrawer suggests that some potential donors had experienced pressure from the recipient and hesitated to donate their kidneys. It also emphasizes the need for repeated education to help potential donors consider the pros and cons of donation sufficiently and the importance of providing an opportunity of voluntary decision without external pressure at any step of donor evaluation. On the other hand, many recipients refused to receive living kidneys from their potential donors, especially their offspring donors because they did not want to give harm to their potential donors, this being the most common cause among the recipient-related factors. Refusal of donor family consent also contributed to kidney donation discontinuation because the donor family did not want their offspring or spouse to experience potential harm by donating their kidneys. Overall, these results demonstrate that non-biomedical factors, in addition to biomedical factors, play an important role in kidney donation discontinuation. In US, home-based education program of both recipients and their potential donors was reported to increase living donor kidney transplantation and living donor evaluation^36^. Adapted home-based family education on living donation in Netherlands was reported to help well-informed decision making and promote living donor kidney transplantation^37^. Therefore, sufficient education of potential donors and recipients along with their family before donor evaluation testing could be helpful to enhance efficiency of LDKT process, and development of various education program including home-based education program is important to promote LDKT in Korea.

Previous studies on living kidney donors have primarily focused on absolute and relative contraindication criteria to minimize a donor risk. The barriers of potential living kidney donors have not been sufficiently investigated yet. This study is expected to contribute to improvement of the LDKT preparation process by helping the medical staff understand causes of donation discontinuation more systematically as well as to provide objective data to potential donors and recipients for their better preparation and for informed decision making.

In conclusion, nearly half of the potential living donors did not proceed to living donor kidney transplantation. Donor-related factors were the most common causes of donation discontinuation, followed by immunologic or size mismatch between donors and recipients and recipient-related factors. In addition to biomedical factors, non-biomedical factors also played an important role in donation discontinuation. Although most donation discontinuation cases occurred at the first step, withdrawal of donation consent occurred primarily at second step. Based on these data, we will strengthen our education program about living donation to reduce non-biomedical factors. Understanding of barriers to living kidney donation could contribute to more efficient and safer living kidney donation.

## Methods

Medical records of 1273 potential recipients and potential 1658 donors who visited Seoul National University Hospital between 2010 and 2017 for living donor kidney transplantation were retrospectively analyzed to investigate the causes of kidney donation discontinuation during the donor work up process.

The donor evaluation consists of three steps. The first step is to check the contraindications for living kidney donation through history taking and physical examination by physicians and basic laboratory tests including blood test, urine test, simple chest radiography, electrocardiogram, ABO/Rh blood typing and cross-match test with the recipient at an out-patient clinic. And physicians give an education to both recipients and their potential donors about outline of kidney transplantation, and advantage and disadvantage of living donor kidney transplantation. After fulfilling the first step, the second step of donor evaluation in either out-patient clinic or on an in-patient basis, includes viral marker study, human leukocyte antigen (HLA) typing, endoscopy, mammography, simple imaging of kidney, ureter, and bladder (KUB), paranasal sinus (PNS) series, computed tomography (CT) angiography, cardiac evaluation according to risk profiles, and several consultations. Potential donors with hypertension receive fundoscopic examination and echocardiography to exclude target organ damage of hypertension. Donors with a family history of DM or those with notably high blood sugar levels during the first step should receive oral glucose tolerance test (OGTT). In case of borderline eGFR, direct GFR measurement using ^51^Cr-ethylenediamine tetraacetic acid (EDTA) or renal scan is performed. Dental and otorhinolaryngological evaluations to check malignancy and active infection are performed by consultation. A psychiatric consultation is essential to check the presence of anxiety, stress, and major psychotic disorders and spontaneity of donation consent. Interviews with a transplant coordinator and a medical social welfare team are also conducted to evaluate spontaneity of donation consent and psychosocial problems. In the third step, a joint meeting of the transplantation team including physicians, surgeons, and coordinators finally determine suitability of potential donors as living kidney donors and the side from which the kidney will be procured from donors (Fig. 1).

This study was approved by the Institutional Review Board of the Seoul National University Hospital (H-2002-162-1106). Informed consent was waived by the Institutional Review Board. And this study was performed in accordance with the Declaration of Helsinki of 2000 and the Declaration of Istanbul of 2008.

Categorical variables are expressed as frequency or percentage, and continuous variables are expressed as mean ± standard deviation. Data was analyzed using analysis of variance or Mann–Whitney *U* test for continuous variables, as appropriate. Patient survival and death-censored graft survival were estimated using the Kaplan–Meier method. Statistical analysis was performed using SPSS version 20.0 (SPSS Inc, Chicago, IL).
